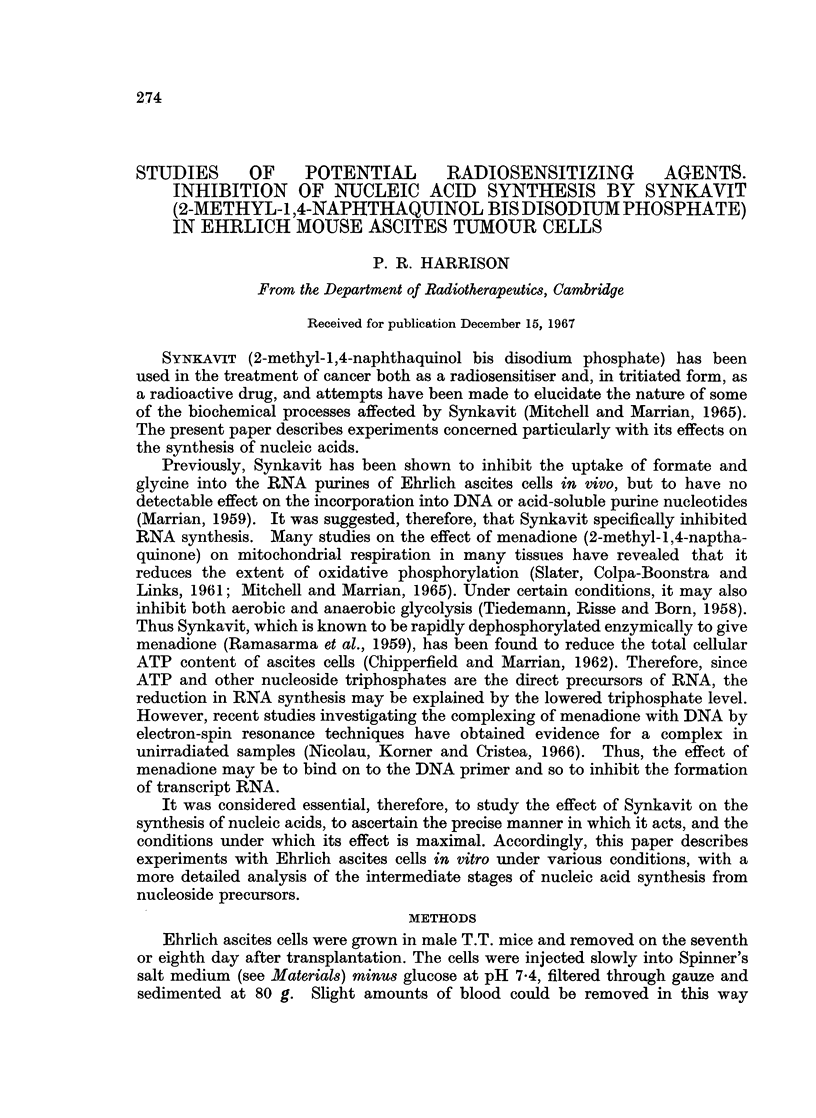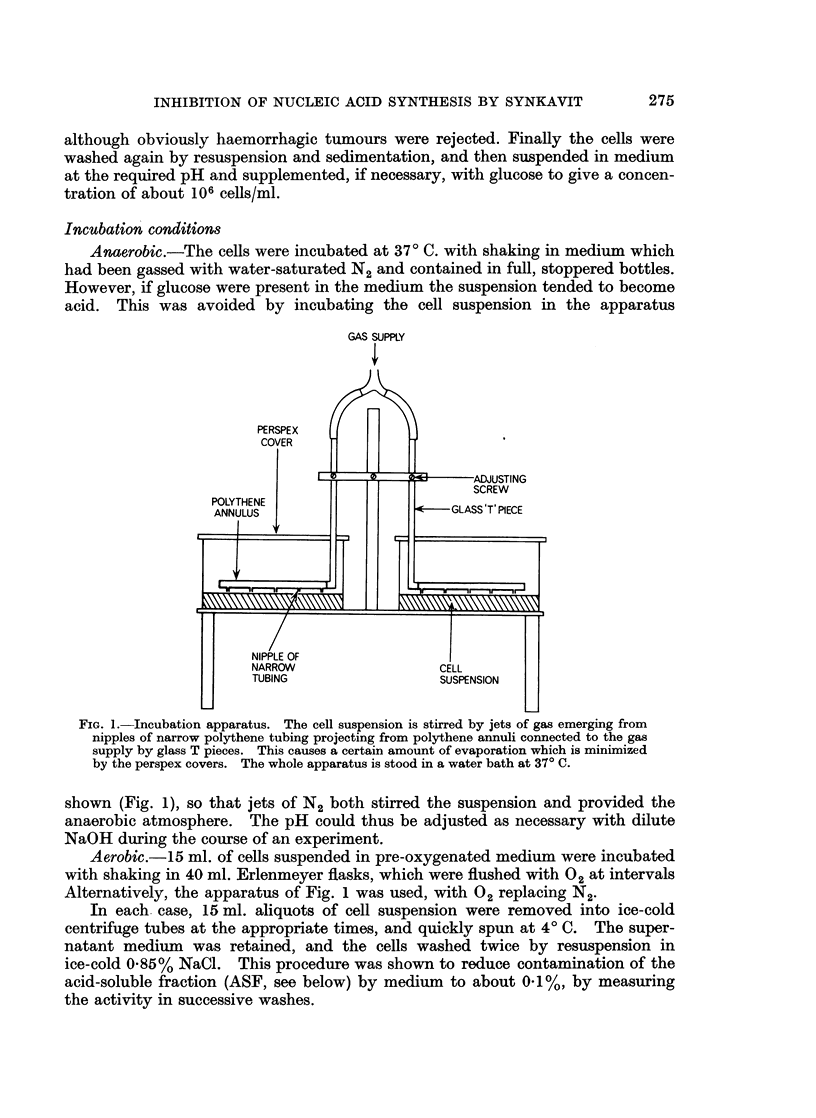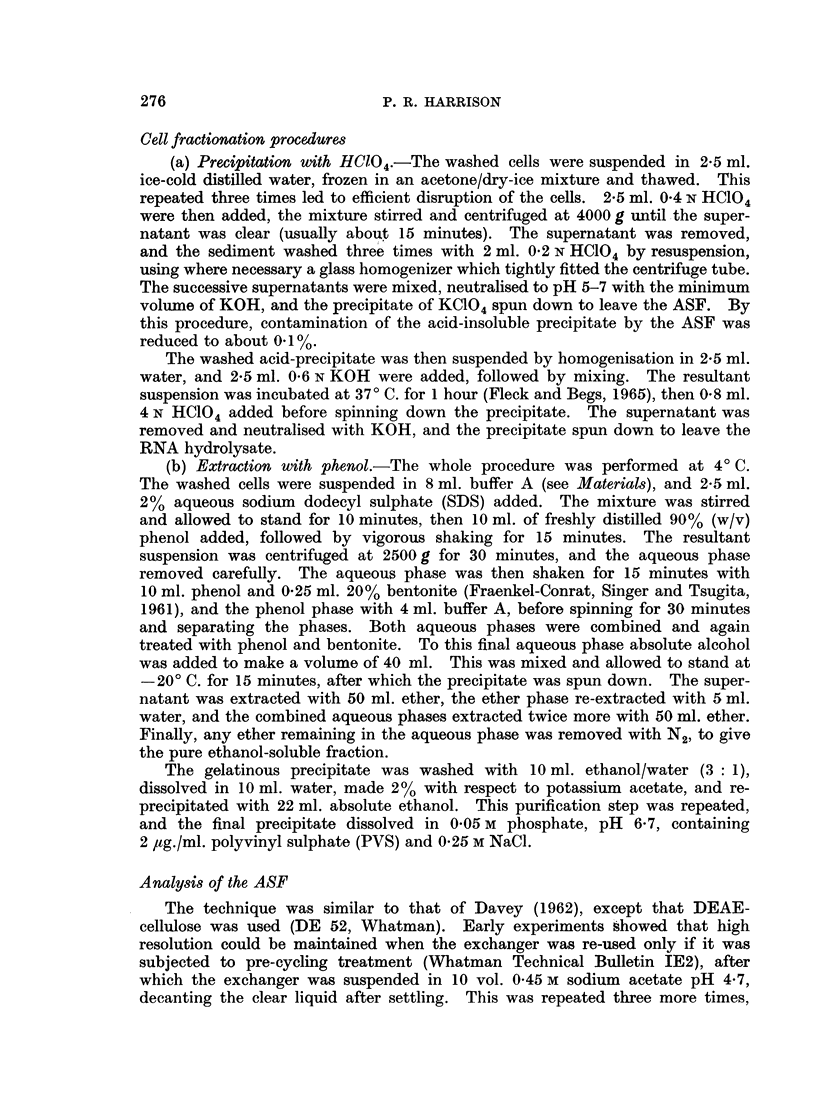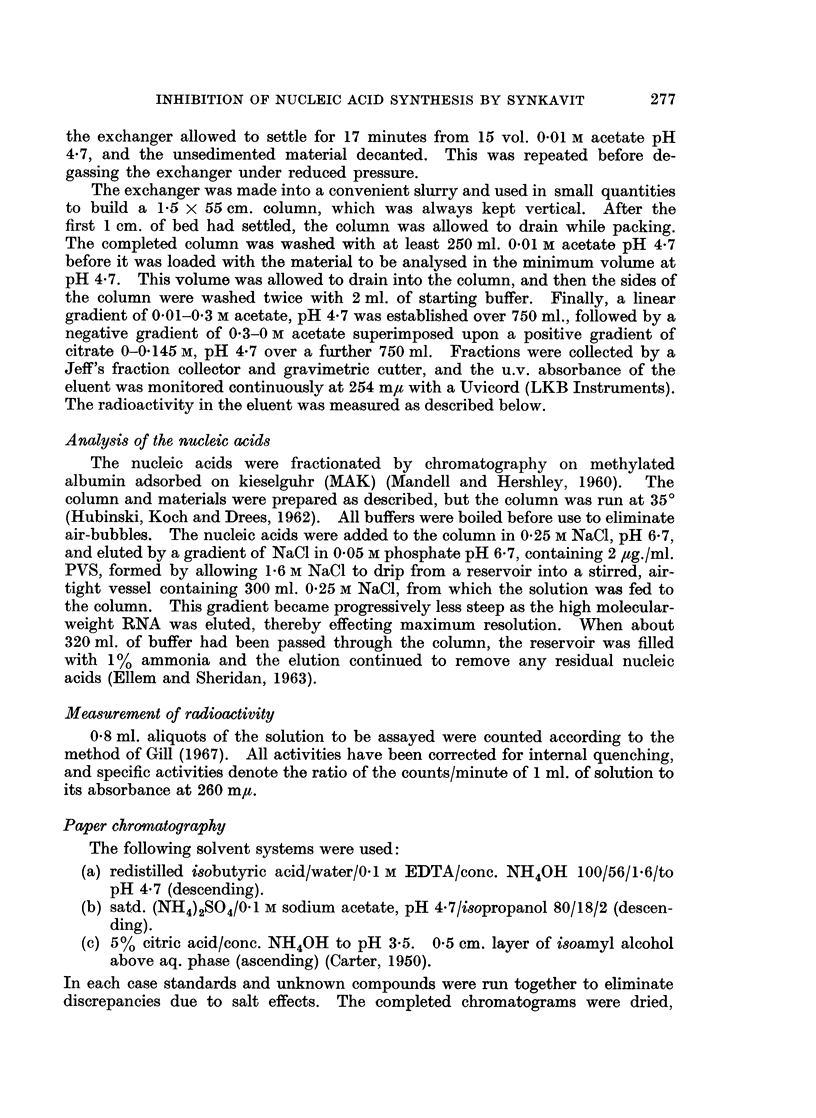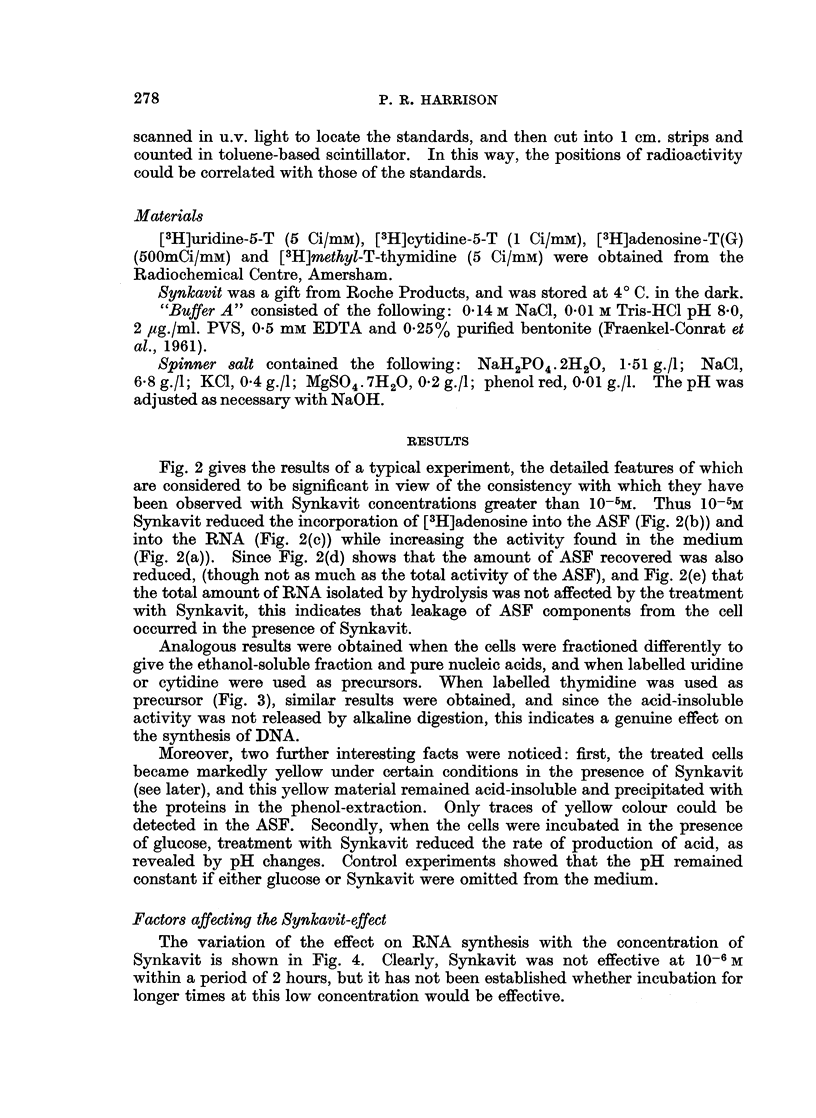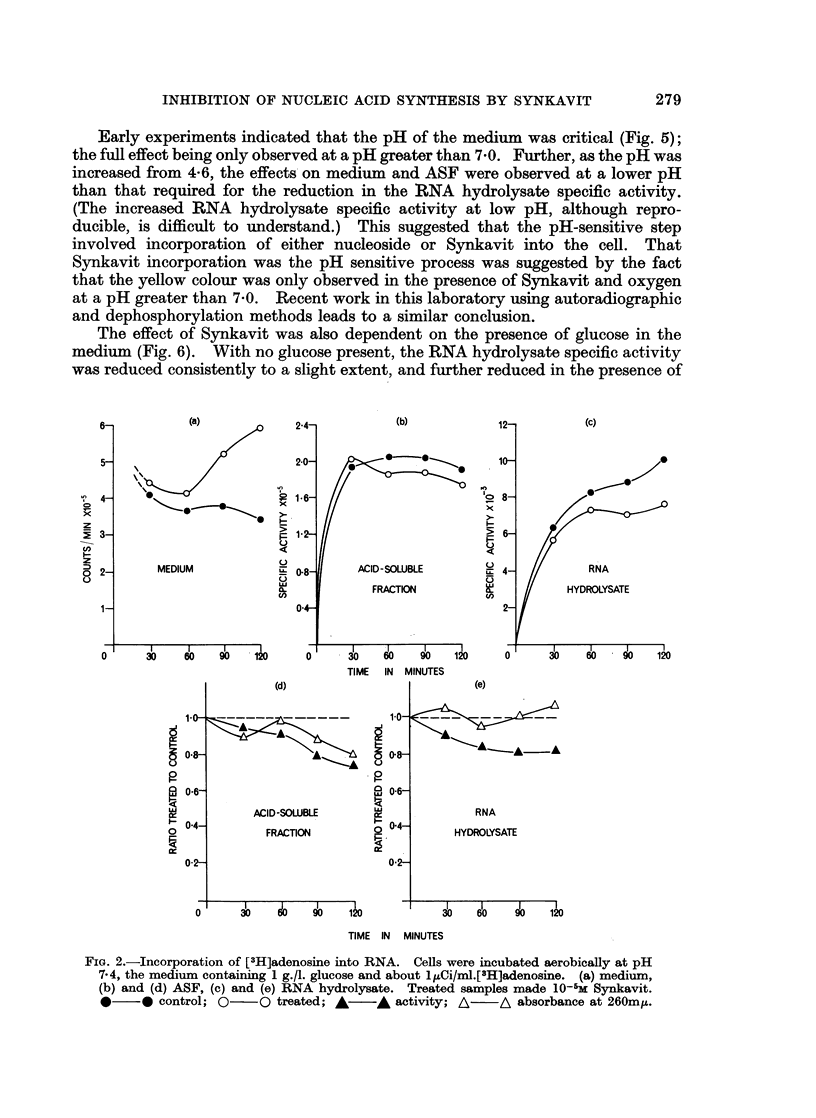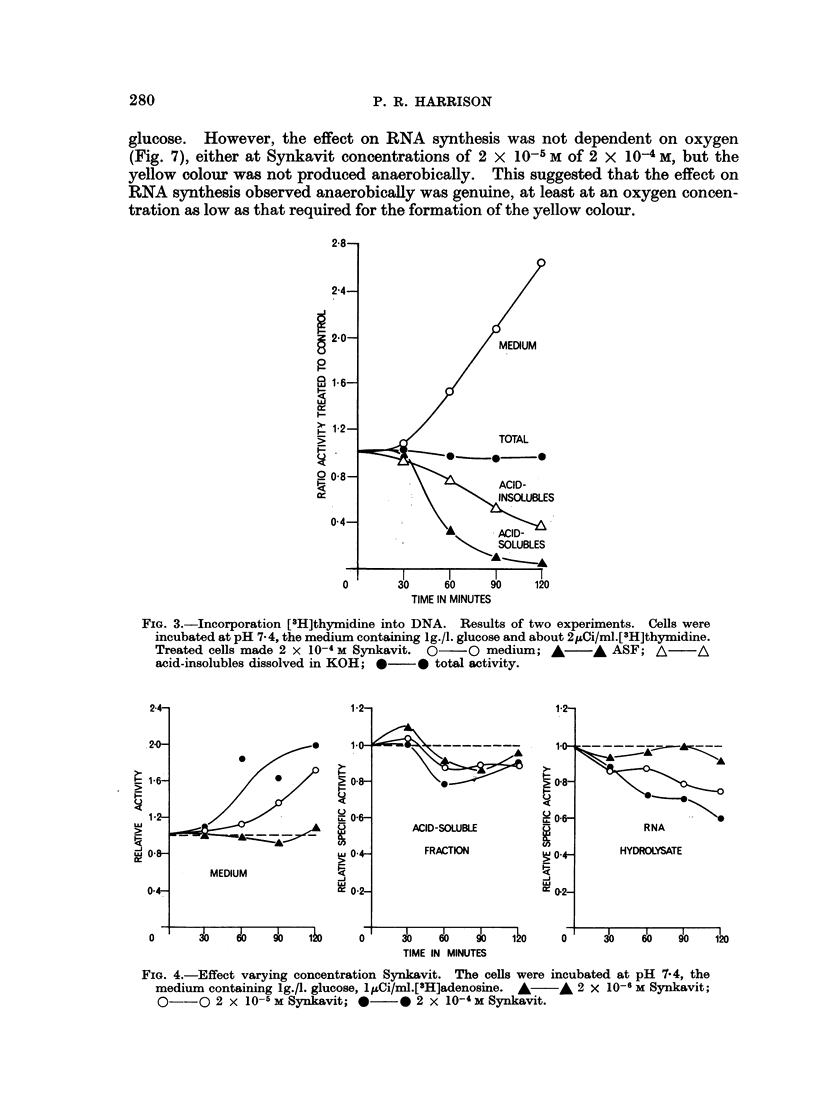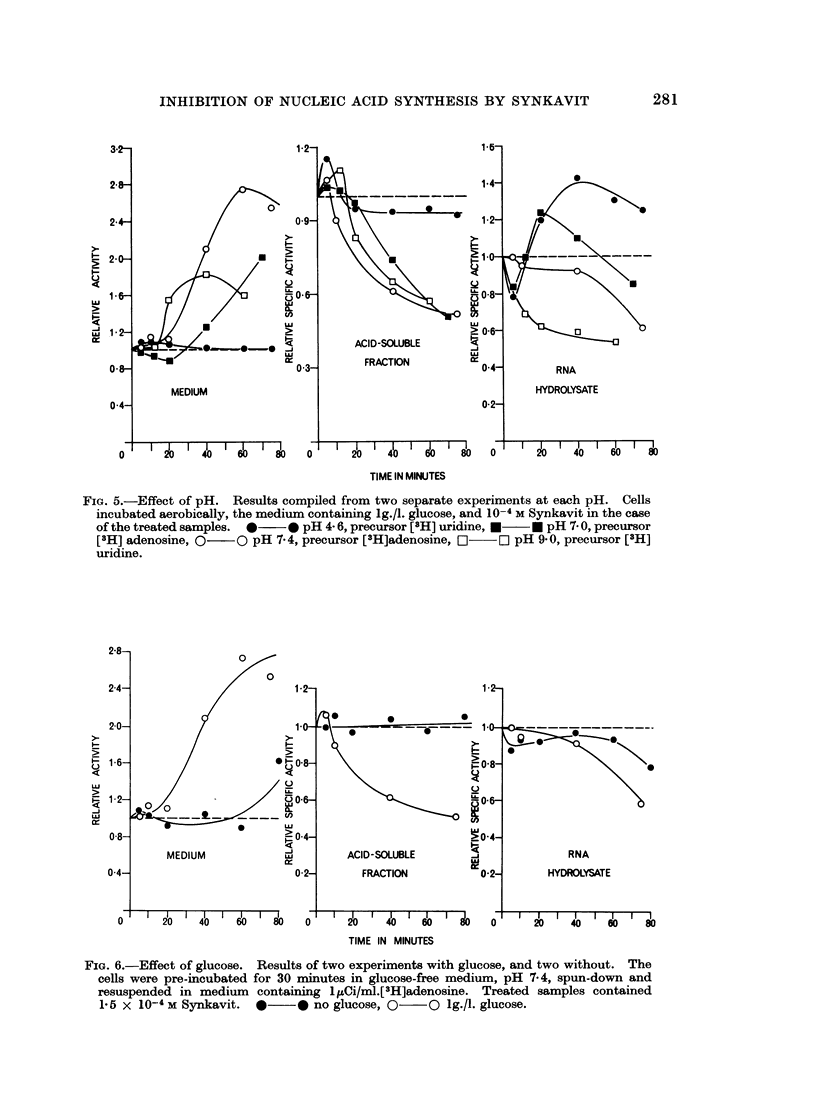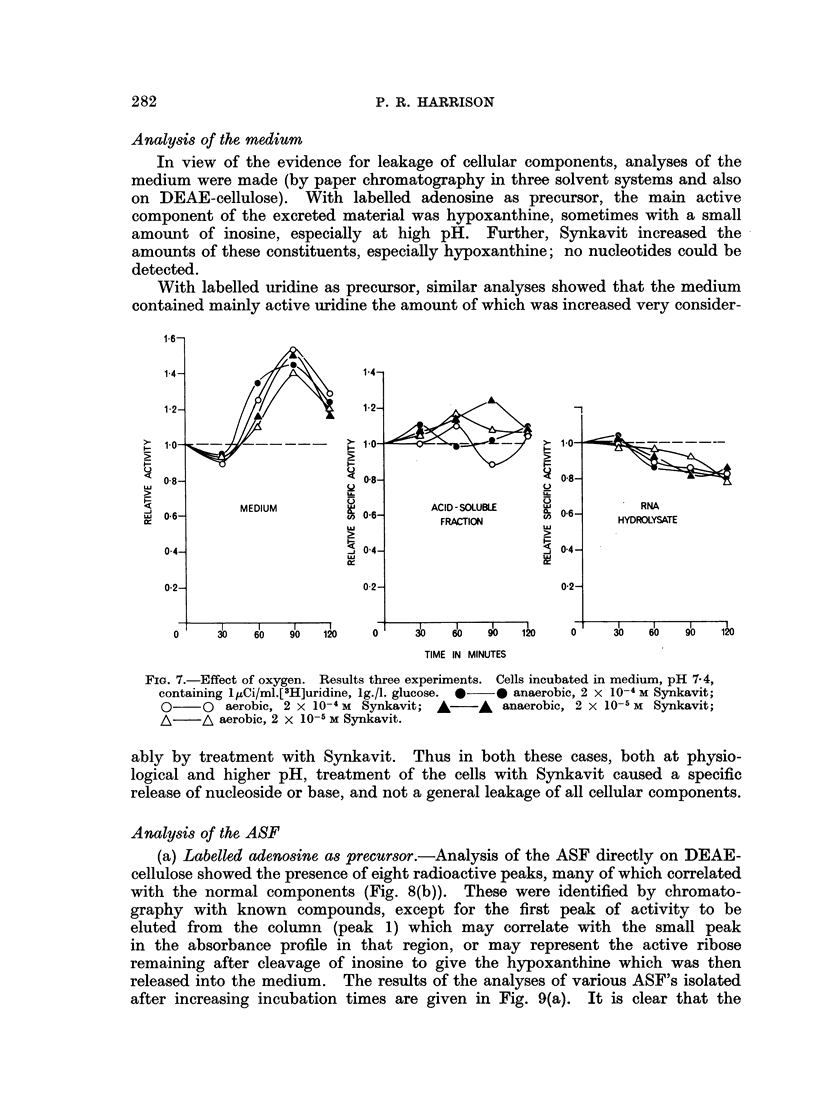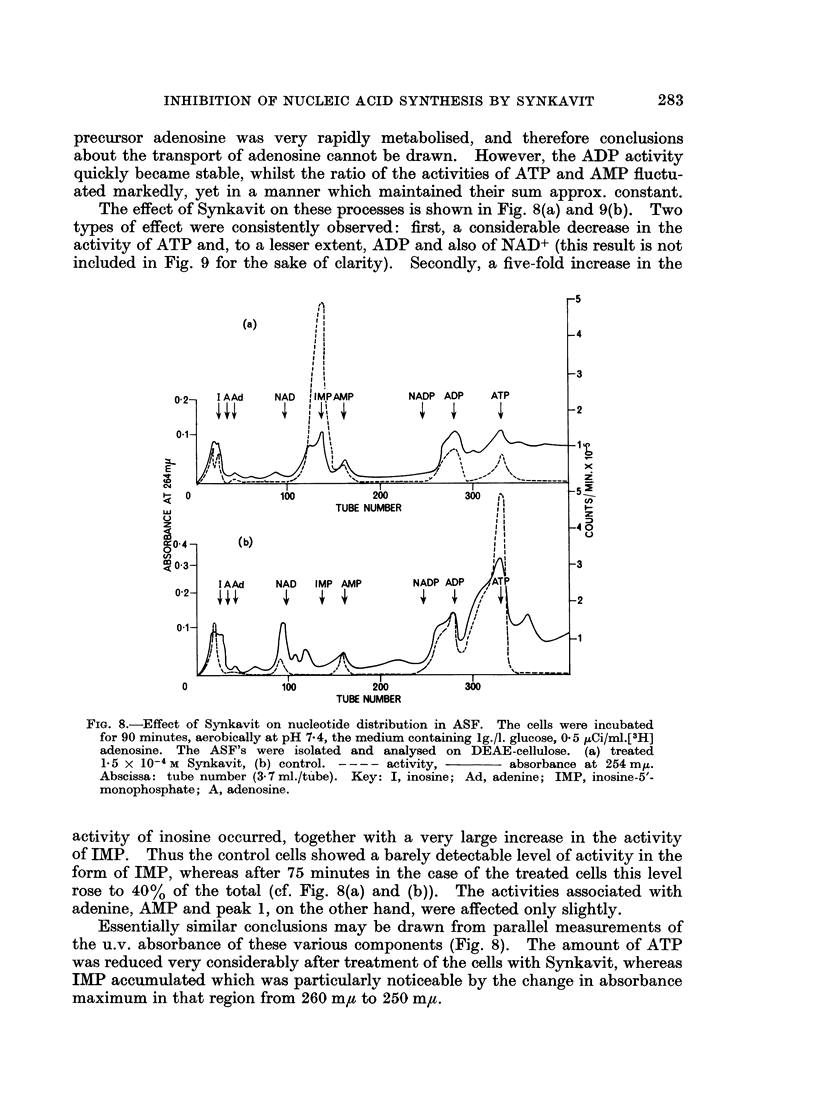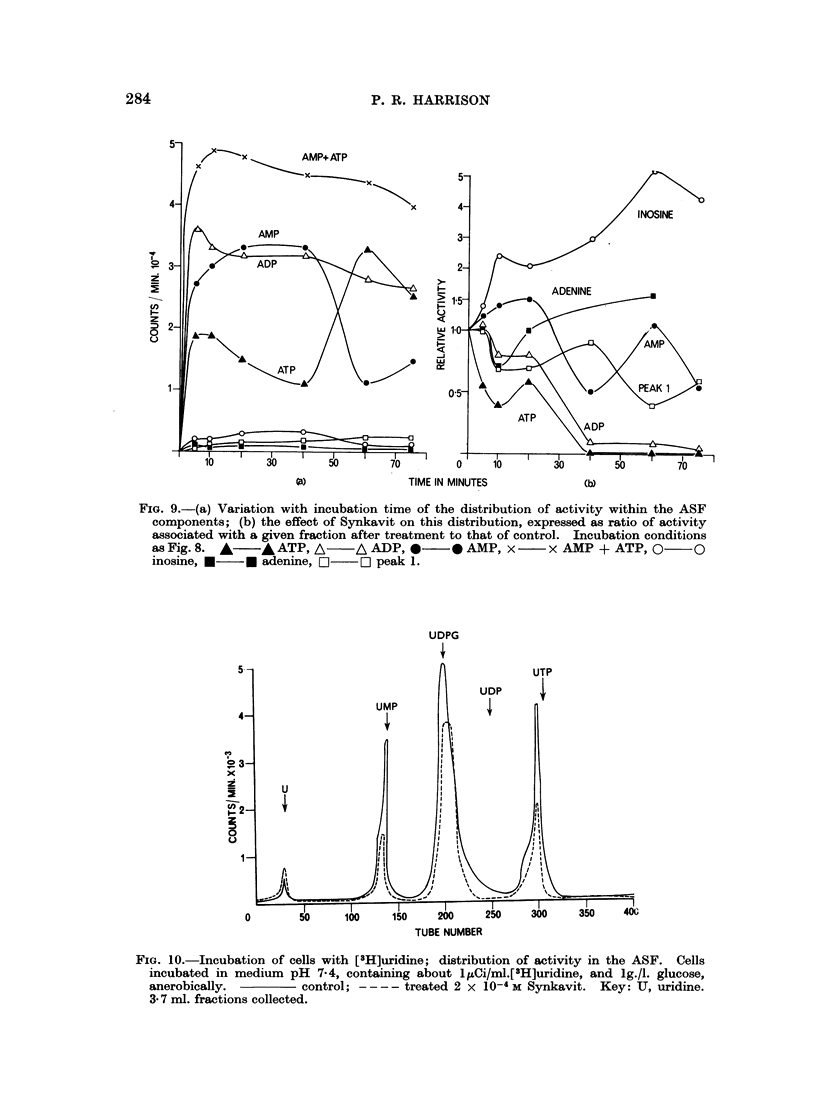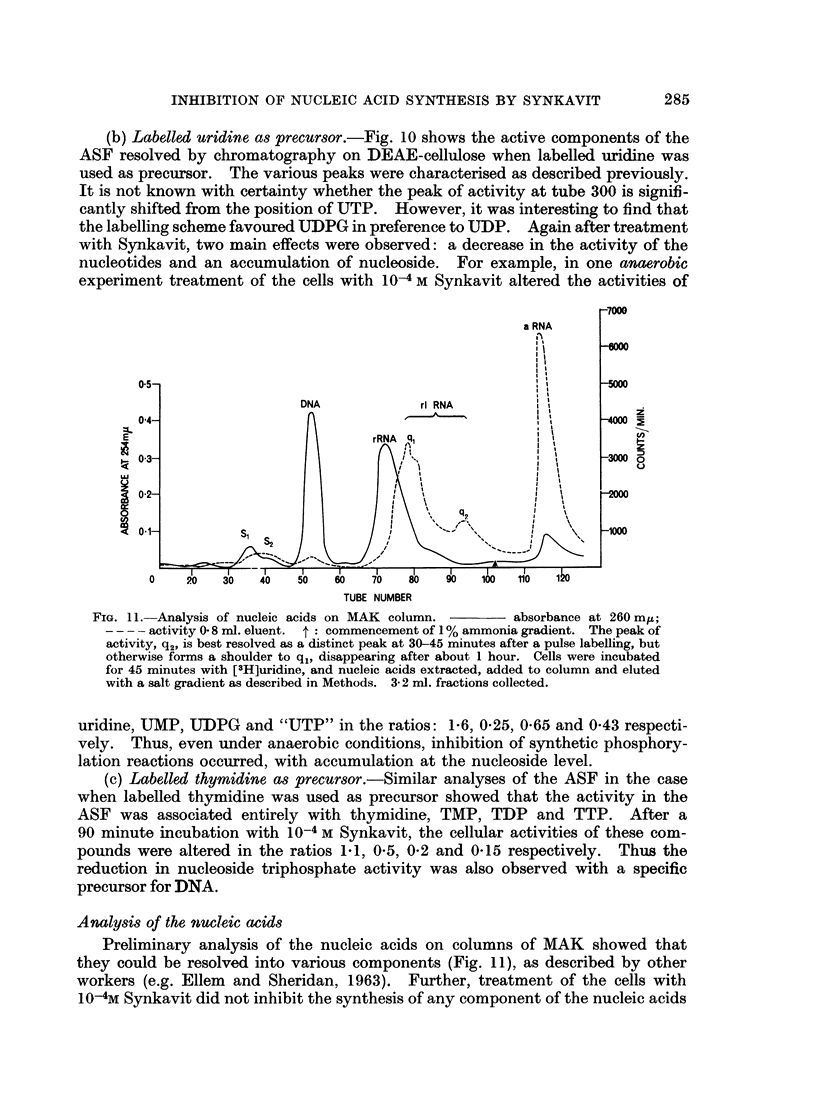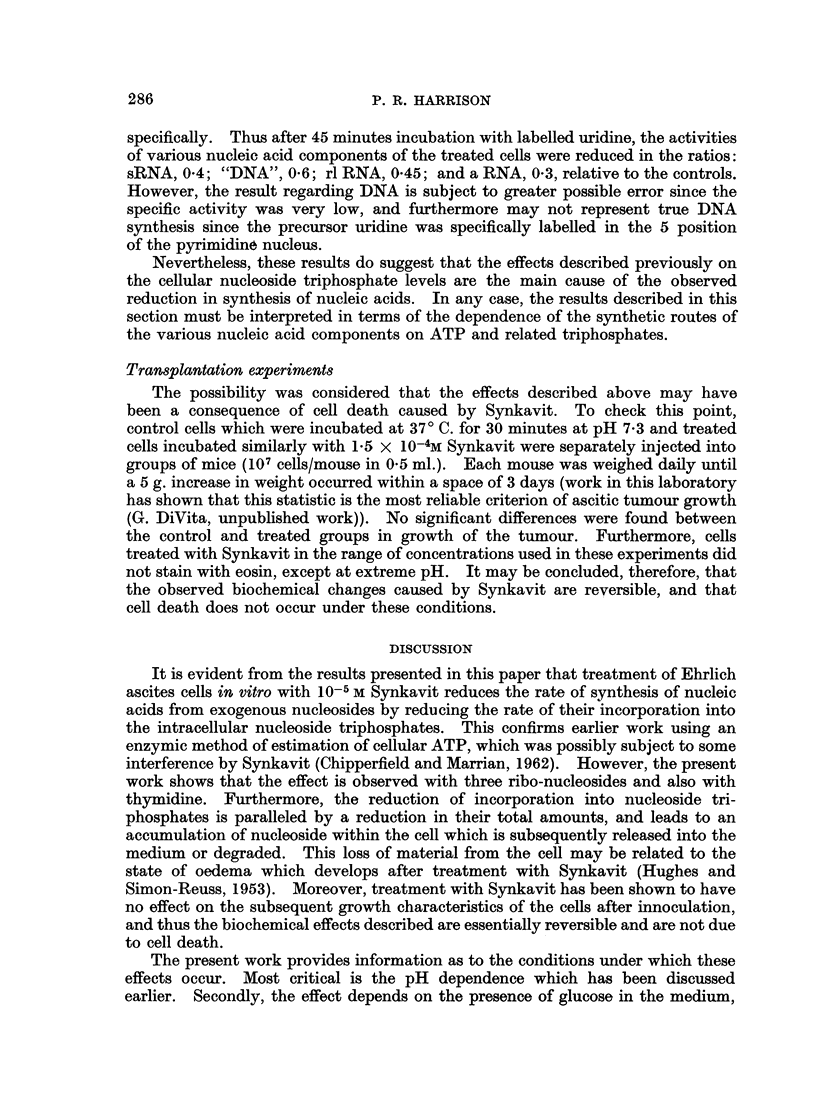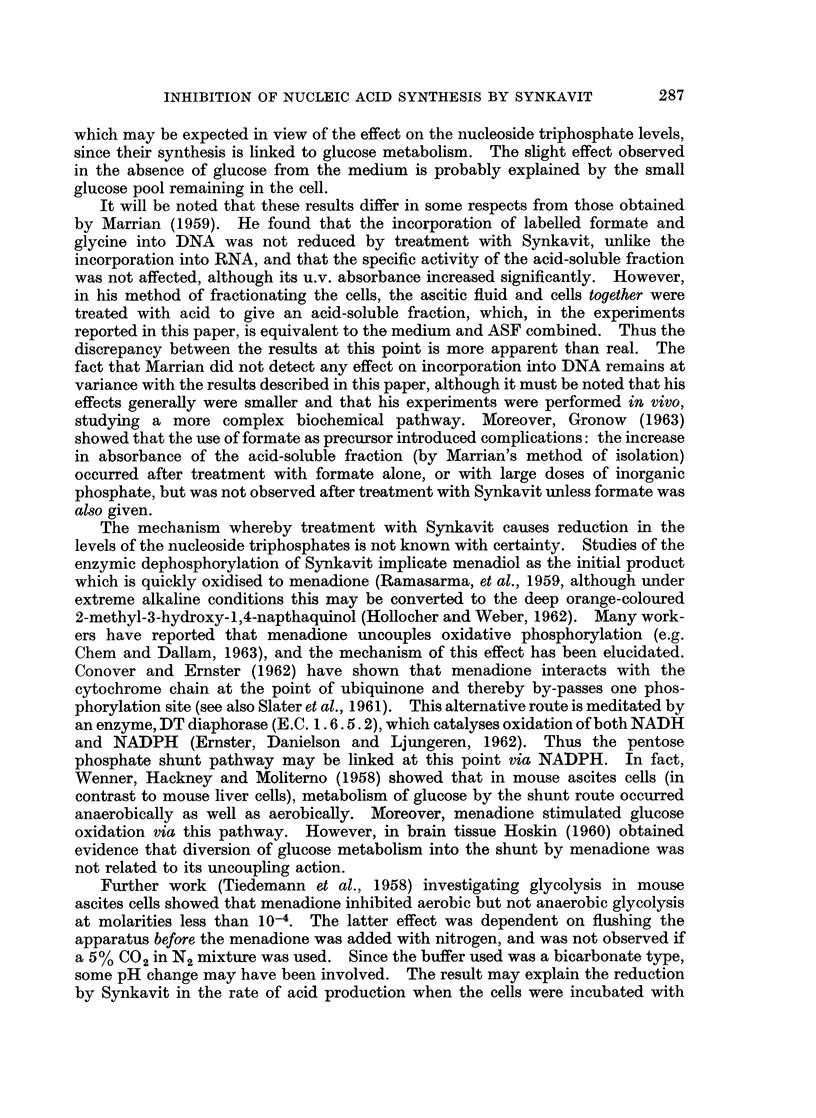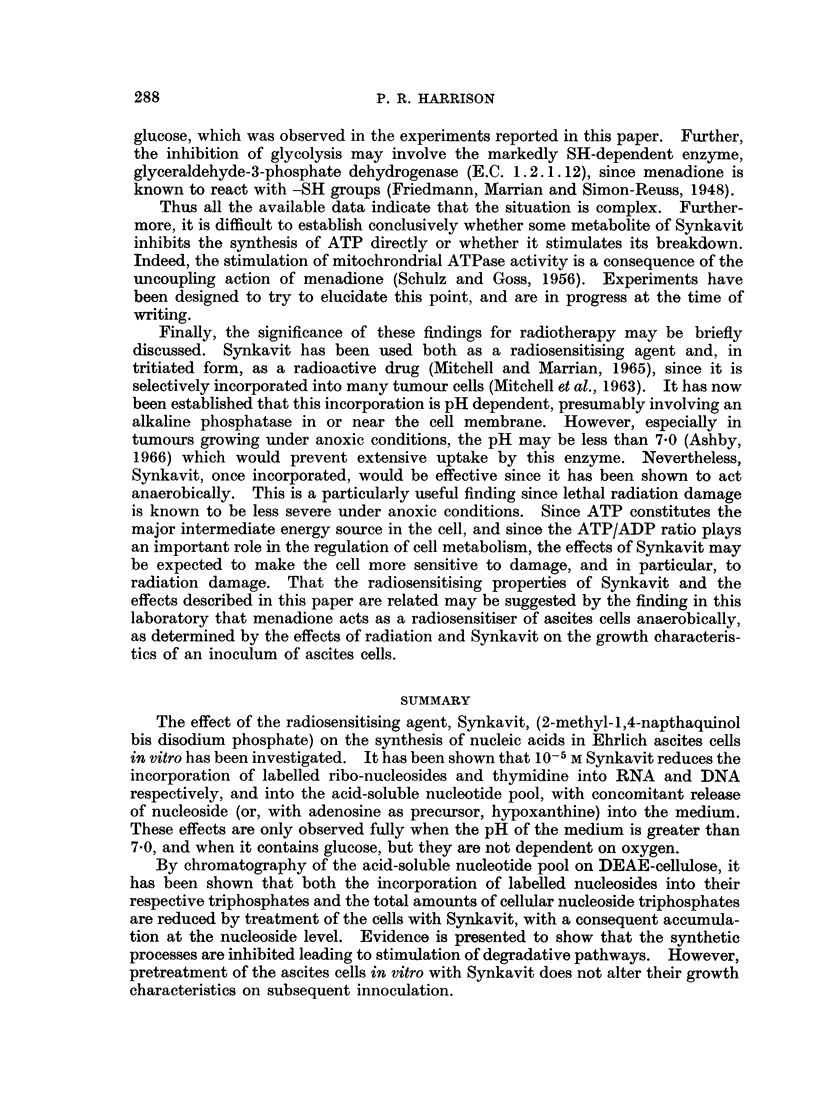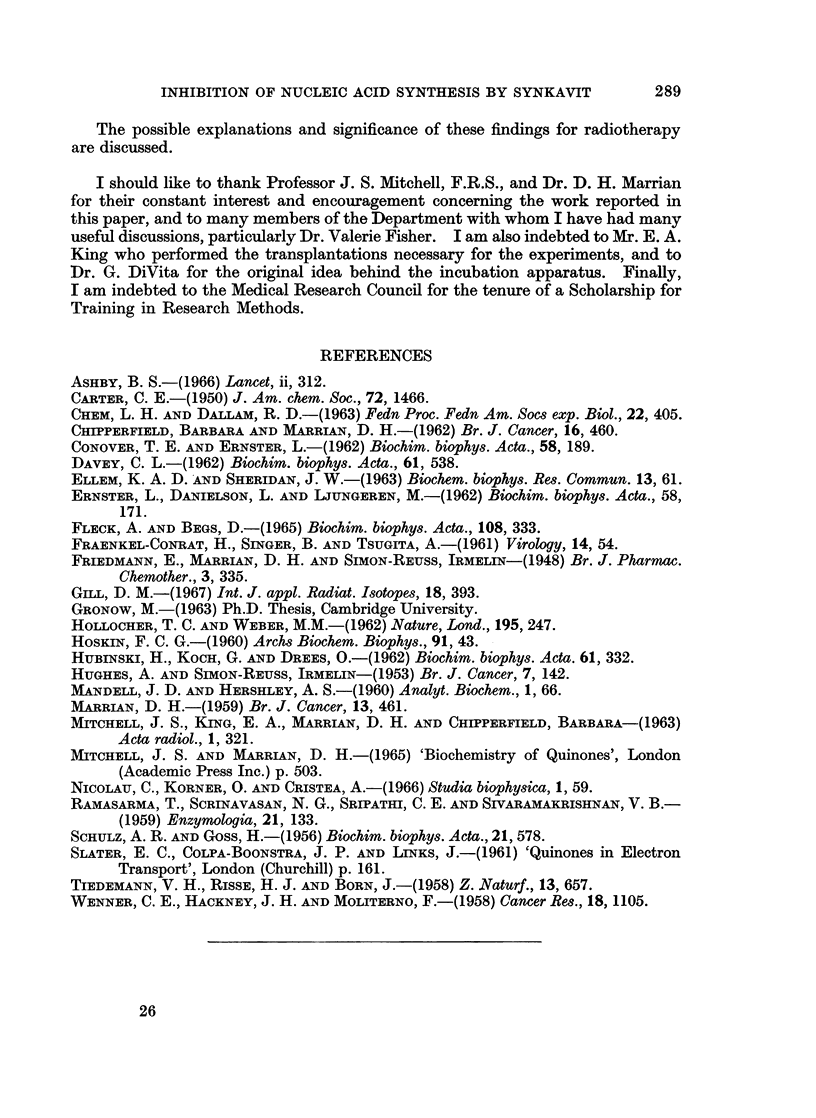# Studies of potential radiosensitizing agents. Inhibition of nucleic acid synthesis by Synkavit (2-methyl-1,4-naphthaquinol bis disodium phosphate) in Ehrlich mouse ascites tumour cells.

**DOI:** 10.1038/bjc.1968.35

**Published:** 1968-06

**Authors:** P. R. Harrison


### STUDIES OF POTENTIAL RADIOSENSITIZING AGENTS. INHIBITION OF NUCLEIC ACID SYNTHESIS BY SYNKAVIT (2-METHYL-1,4-NAPHTHAQUINOL BIS DISODIUM PHOSPHATE) IN EHRLICH MOUSE ASCITES TUMOUR CELLS

P. R. HARRISON

*From the Department of Radiotherapeutics, Cambridge*

Received for publication December 15, 1967

SYNKAVIT (2-methyl-1,4-naphthaquinol bis disodium phosphate) has been used in the treatment of cancer both as a radiosensitiser and, in tritiated form, as a radioactive drug, and attempts have been made to elucidate the nature of some of the biochemical processes affected by Synkavit (Mitchell and Marrian, 1965). The present paper describes experiments concerned particularly with its effects on the synthesis of nucleic acids.

Previously, Synkavit has been shown to inhibit the uptake of formate and glycine into the RNA purines of Ehrlich ascites cells *in vivo*, but to have no detectable effect on the incorporation into DNA or acid-soluble purine nucleotides (Marrian, 1959). It was suggested, therefore, that Synkavit specifically inhibited RNA synthesis. Many studies on the effect of menadione (2-methyl-1,4-naptha-quinone) on mitochondrial respiration in many tissues have revealed that it reduces the extent of oxidative phosphorylation (Slater, Colpa-Boonstra and Links, 1961; Mitchell and Marrian, 1965). Under certain conditions, it may also inhibit both aerobic and anaerobic glycolysis (Tiedemann, Risse and Born, 1958). Thus Synkavit, which is known to be rapidly dephosphorylated enzymically to give menadione (Ramasarma *et al.*, 1959), has been found to reduce the total cellular ATP content of ascites cells (Chipperfield and Marrian, 1962). Therefore, since ATP and other nucleoside triphosphates are the direct precursors of RNA, the reduction in RNA synthesis may be explained by the lowered triphosphate level. However, recent studies investigating the complexing of menadione with DNA by electron-spin resonance techniques have obtained evidence for a complex in unirradiated samples (Nicolau, Korner and Cristea, 1966). Thus, the effect of menadione may be to bind on to the DNA primer and so to inhibit the formation of transcript RNA.

It was considered essential, therefore, to study the effect of Synkavit on the synthesis of nucleic acids, to ascertain the precise manner in which it acts, and the conditions under which its effect is maximal. Accordingly, this paper describes experiments with Ehrlich ascites cells *in vitro* under various conditions, with a more detailed analysis of the intermediate stages of nucleic acid synthesis from nucleoside precursors.

### METHODS

Ehrlich ascites cells were grown in male T.T. mice and removed on the seventh or eighth day after transplantation. The cells were injected slowly into Spinner's salt medium (see *Materials*) *minus* glucose at pH 7·4, filtered through gauze and sedimented at 80 *g*. Slight amounts of blood could be removed in this way although obviously haemorrhagic tumours were rejected. Finally the cells were washed again by resuspension and sedimentation, and then suspended in medium at the required pH and supplemented, if necessary, with glucose to give a concentration of about $10^6$ cells/ml.

*Incubation conditions*

*Anaerobic.*—The cells were incubated at 37° C. with shaking in medium which had been gassed with water-saturated $N_2$ and contained in full, stoppered bottles. However, if glucose were present in the medium the suspension tended to become acid. This was avoided by incubating the cell suspension in the apparatus

FIG. 1.—Incubation apparatus. The cell suspension is stirred by jets of gas emerging from nipples of narrow polythene tubing projecting from polythene annuli connected to the gas supply by glass T pieces. This causes a certain amount of evaporation which is minimized by the perspex covers. The whole apparatus is stood in a water bath at 37° C.

shown (Fig. 1), so that jets of $N_2$ both stirred the suspension and provided the anaerobic atmosphere. The pH could thus be adjusted as necessary with dilute NaOH during the course of an experiment.

*Aerobic.*—15 ml. of cells suspended in pre-oxygenated medium were incubated with shaking in 40 ml. Erlenmeyer flasks, which were flushed with $O_2$ at intervals Alternatively, the apparatus of Fig. 1 was used, with $O_2$ replacing $N_2$.

In each case, 15 ml. aliquots of cell suspension were removed into ice-cold centrifuge tubes at the appropriate times, and quickly spun at 4° C. The supernatant medium was retained, and the cells washed twice by resuspension in ice-cold 0·85% NaCl. This procedure was shown to reduce contamination of the acid-soluble fraction (ASF, see below) by medium to about 0·1%, by measuring the activity in successive washes.

*Cell fractionation procedures*

(a) *Precipitation with $HClO_4$*.—The washed cells were suspended in 2·5 ml. ice-cold distilled water, frozen in an acetone/dry-ice mixture and thawed. This repeated three times led to efficient disruption of the cells. 2·5 ml. 0·4 N $HClO_4$ were then added, the mixture stirred and centrifuged at 4000 *g* until the supernatant was clear (usually about 15 minutes). The supernatant was removed, and the sediment washed three times with 2 ml. 0·2 N $HClO_4$ by resuspension, using where necessary a glass homogenizer which tightly fitted the centrifuge tube. The successive supernatants were mixed, neutralised to pH 5–7 with the minimum volume of KOH, and the precipitate of $KClO_4$ spun down to leave the ASF. By this procedure, contamination of the acid-insoluble precipitate by the ASF was reduced to about 0·1%.

The washed acid-precipitate was then suspended by homogenisation in 2·5 ml. water, and 2·5 ml. 0·6 N KOH were added, followed by mixing. The resultant suspension was incubated at 37° C. for 1 hour (Fleck and Begs, 1965), then 0·8 ml. 4 N $HClO_4$ added before spinning down the precipitate. The supernatant was removed and neutralised with KOH, and the precipitate spun down to leave the RNA hydrolysate.

(b) *Extraction with phenol*.—The whole procedure was performed at 4° C. The washed cells were suspended in 8 ml. buffer A (see *Materials*), and 2·5 ml. 2% aqueous sodium dodecyl sulphate (SDS) added. The mixture was stirred and allowed to stand for 10 minutes, then 10 ml. of freshly distilled 90% (w/v) phenol added, followed by vigorous shaking for 15 minutes. The resultant suspension was centrifuged at 2500 *g* for 30 minutes, and the aqueous phase removed carefully. The aqueous phase was then shaken for 15 minutes with 10 ml. phenol and 0·25 ml. 20% bentonite (Fraenkel-Conrat, Singer and Tsugita, 1961), and the phenol phase with 4 ml. buffer A, before spinning for 30 minutes and separating the phases. Both aqueous phases were combined and again treated with phenol and bentonite. To this final aqueous phase absolute alcohol was added to make a volume of 40 ml. This was mixed and allowed to stand at −20° C. for 15 minutes, after which the precipitate was spun down. The supernatant was extracted with 50 ml. ether, the ether phase re-extracted with 5 ml. water, and the combined aqueous phases extracted twice more with 50 ml. ether. Finally, any ether remaining in the aqueous phase was removed with $N_2$, to give the pure ethanol-soluble fraction.

The gelatinous precipitate was washed with 10 ml. ethanol/water (3 : 1), dissolved in 10 ml. water, made 2% with respect to potassium acetate, and re-precipitated with 22 ml. absolute ethanol. This purification step was repeated, and the final precipitate dissolved in 0·05 M phosphate, pH 6·7, containing 2 $\mu$g./ml. polyvinyl sulphate (PVS) and 0·25 M NaCl.

*Analysis of the ASF*

The technique was similar to that of Davey (1962), except that DEAE-cellulose was used (DE 52, Whatman). Early experiments showed that high resolution could be maintained when the exchanger was re-used only if it was subjected to pre-cycling treatment (Whatman Technical Bulletin IE2), after which the exchanger was suspended in 10 vol. 0·45 M sodium acetate pH 4·7, decanting the clear liquid after settling. This was repeated three more times, the exchanger allowed to settle for 17 minutes from 15 vol. 0·01 M acetate pH 4·7, and the unsedimented material decanted. This was repeated before degassing the exchanger under reduced pressure.

The exchanger was made into a convenient slurry and used in small quantities to build a 1·5 × 55 cm. column, which was always kept vertical. After the first 1 cm. of bed had settled, the column was allowed to drain while packing. The completed column was washed with at least 250 ml. 0·01 M acetate pH 4·7 before it was loaded with the material to be analysed in the minimum volume at pH 4·7. This volume was allowed to drain into the column, and then the sides of the column were washed twice with 2 ml. of starting buffer. Finally, a linear gradient of 0·01–0·3 M acetate, pH 4·7 was established over 750 ml., followed by a negative gradient of 0·3–0 M acetate superimposed upon a positive gradient of citrate 0–0·145 M, pH 4·7 over a further 750 ml. Fractions were collected by a Jeff's fraction collector and gravimetric cutter, and the u.v. absorbance of the eluent was monitored continuously at 254 m$\mu$ with a Uvicord (LKB Instruments). The radioactivity in the eluent was measured as described below.

*Analysis of the nucleic acids*

The nucleic acids were fractionated by chromatography on methylated albumin adsorbed on kieselguhr (MAK) (Mandell and Hershley, 1960). The column and materials were prepared as described, but the column was run at 35° (Hubinski, Koch and Drees, 1962). All buffers were boiled before use to eliminate air-bubbles. The nucleic acids were added to the column in 0·25 M NaCl, pH 6·7, and eluted by a gradient of NaCl in 0·05 M phosphate pH 6·7, containing 2 $\mu$g./ml. PVS, formed by allowing 1·6 M NaCl to drip from a reservoir into a stirred, airtight vessel containing 300 ml. 0·25 M NaCl, from which the solution was fed to the column. This gradient became progressively less steep as the high molecular-weight RNA was eluted, thereby effecting maximum resolution. When about 320 ml. of buffer had been passed through the column, the reservoir was filled with 1% ammonia and the elution continued to remove any residual nucleic acids (Ellem and Sheridan, 1963).

*Measurement of radioactivity*

0·8 ml. aliquots of the solution to be assayed were counted according to the method of Gill (1967). All activities have been corrected for internal quenching, and specific activities denote the ratio of the counts/minute of 1 ml. of solution to its absorbance at 260 m$\mu$.

*Paper chromatography*

The following solvent systems were used:

(a) redistilled *iso*butyric acid/water/0·1 M EDTA/conc. $NH_4OH$ 100/56/1·6/to pH 4·7 (descending).
(b) satd. $(NH_4)_2SO_4$/0·1 M sodium acetate, pH 4·7/*iso*propanol 80/18/2 (descending).
(c) 5% citric acid/conc. $NH_4OH$ to pH 3·5. 0·5 cm. layer of *iso*amyl alcohol above aq. phase (ascending) (Carter, 1950).

In each case standards and unknown compounds were run together to eliminate discrepancies due to salt effects. The completed chromatograms were dried, scanned in u.v. light to locate the standards, and then cut into 1 cm. strips and counted in toluene-based scintillator. In this way, the positions of radioactivity could be correlated with those of the standards.

### *Materials*

[$^3$H]uridine-5-T (5 Ci/mM), [$^3$H]cytidine-5-T (1 Ci/mM), [$^3$H]adenosine-T(G) (500mCi/mM) and [$^3$H]*methyl*-T-thymidine (5 Ci/mM) were obtained from the Radiochemical Centre, Amersham.

*Synkavit* was a gift from Roche Products, and was stored at 4° C. in the dark.

"*Buffer A*" consisted of the following: 0·14 M NaCl, 0·01 M Tris-HCl pH 8·0, 2 $\mu$g./ml. PVS, 0·5 mM EDTA and 0·25% purified bentonite (Fraenkel-Conrat *et al.*, 1961).

*Spinner salt* contained the following: $NaH_2PO_4.2H_2O$, 1·51 g./l; NaCl, 6·8 g./l; KCl, 0·4 g./l; $MgSO_4.7H_2O$, 0·2 g./l; phenol red, 0·01 g./l. The pH was adjusted as necessary with NaOH.

### RESULTS

Fig. 2 gives the results of a typical experiment, the detailed features of which are considered to be significant in view of the consistency with which they have been observed with Synkavit concentrations greater than $10^{-5}$M. Thus $10^{-5}$M Synkavit reduced the incorporation of [$^3$H]adenosine into the ASF (Fig. 2(b)) and into the RNA (Fig. 2(c)) while increasing the activity found in the medium (Fig. 2(a)). Since Fig. 2(d) shows that the amount of ASF recovered was also reduced, (though not as much as the total activity of the ASF), and Fig. 2(e) that the total amount of RNA isolated by hydrolysis was not affected by the treatment with Synkavit, this indicates that leakage of ASF components from the cell occurred in the presence of Synkavit.

Analogous results were obtained when the cells were fractioned differently to give the ethanol-soluble fraction and pure nucleic acids, and when labelled uridine or cytidine were used as precursors. When labelled thymidine was used as precursor (Fig. 3), similar results were obtained, and since the acid-insoluble activity was not released by alkaline digestion, this indicates a genuine effect on the synthesis of DNA.

Moreover, two further interesting facts were noticed: first, the treated cells became markedly yellow under certain conditions in the presence of Synkavit (see later), and this yellow material remained acid-insoluble and precipitated with the proteins in the phenol-extraction. Only traces of yellow colour could be detected in the ASF. Secondly, when the cells were incubated in the presence of glucose, treatment with Synkavit reduced the rate of production of acid, as revealed by pH changes. Control experiments showed that the pH remained constant if either glucose or Synkavit were omitted from the medium.

### *Factors affecting the Synkavit-effect*

The variation of the effect on RNA synthesis with the concentration of Synkavit is shown in Fig. 4. Clearly, Synkavit was not effective at $10^{-6}$ M within a period of 2 hours, but it has not been established whether incubation for longer times at this low concentration would be effective.

Early experiments indicated that the pH of the medium was critical (Fig. 5); the full effect being only observed at a pH greater than 7·0. Further, as the pH was increased from 4·6, the effects on medium and ASF were observed at a lower pH than that required for the reduction in the RNA hydrolysate specific activity. (The increased RNA hydrolysate specific activity at low pH, although reproducible, is difficult to understand.) This suggested that the pH-sensitive step involved incorporation of either nucleoside or Synkavit into the cell. That Synkavit incorporation was the pH sensitive process was suggested by the fact that the yellow colour was only observed in the presence of Synkavit and oxygen at a pH greater than 7·0. Recent work in this laboratory using autoradiographic and dephosphorylation methods leads to a similar conclusion.

The effect of Synkavit was also dependent on the presence of glucose in the medium (Fig. 6). With no glucose present, the RNA hydrolysate specific activity was reduced consistently to a slight extent, and further reduced in the presence of

FIG. 2.—Incorporation of [$^3$H]adenosine into RNA. Cells were incubated aerobically at pH 7·4, the medium containing 1 g./l. glucose and about 1$\mu$Ci/ml.[$^3$H]adenosine. (a) medium, (b) and (d) ASF, (c) and (e) RNA hydrolysate. Treated samples made $10^{-5}$M Synkavit. ●——● control; ○——○ treated; ▲——▲ activity; △——△ absorbance at 260m$\mu$.

glucose. However, the effect on RNA synthesis was not dependent on oxygen (Fig. 7), either at Synkavit concentrations of 2 × $10^{-5}$ M of 2 × $10^{-4}$ M, but the yellow colour was not produced anaerobically. This suggested that the effect on RNA synthesis observed anaerobically was genuine, at least at an oxygen concentration as low as that required for the formation of the yellow colour.

FIG. 3.—Incorporation [$^3$H]thymidine into DNA. Results of two experiments. Cells were incubated at pH 7·4, the medium containing 1g./l. glucose and about 2μCi/ml.[$^3$H]thymidine. Treated cells made 2 × $10^{-4}$ M Synkavit. ○——○ medium; ▲——▲ ASF; △——△ acid-insolubles dissolved in KOH; ●——● total activity.

FIG. 4.—Effect varying concentration Synkavit. The cells were incubated at pH 7·4, the medium containing 1g./l. glucose, 1μCi/ml.[$^3$H]adenosine. ▲——▲ 2 × $10^{-6}$ M Synkavit; ○——○ 2 × $10^{-5}$ M Synkavit; ●——● 2 × $10^{-4}$ M Synkavit.

FIG. 5.—Effect of pH. Results compiled from two separate experiments at each pH. Cells incubated aerobically, the medium containing 1g./l. glucose, and $10^{-4}$ M Synkavit in the case of the treated samples. ●——● pH 4·6, precursor [$^3$H] uridine, ■——■ pH 7·0, precursor [$^3$H] adenosine, ○——○ pH 7·4, precursor [$^3$H]adenosine, □——□ pH 9·0, precursor [$^3$H] uridine.

FIG. 6.—Effect of glucose. Results of two experiments with glucose, and two without. The cells were pre-incubated for 30 minutes in glucose-free medium, pH 7·4, spun-down and resuspended in medium containing 1$\mu$Ci/ml.[$^3$H]adenosine. Treated samples contained $1{\cdot}5 \times 10^{-4}$ M Synkavit. ●——● no glucose, ○——○ 1g./l. glucose.

*Analysis of the medium*

In view of the evidence for leakage of cellular components, analyses of the medium were made (by paper chromatography in three solvent systems and also on DEAE-cellulose). With labelled adenosine as precursor, the main active component of the excreted material was hypoxanthine, sometimes with a small amount of inosine, especially at high pH. Further, Synkavit increased the amounts of these constituents, especially hypoxanthine; no nucleotides could be detected.

With labelled uridine as precursor, similar analyses showed that the medium contained mainly active uridine the amount of which was increased very consider-

FIG. 7.—Effect of oxygen. Results three experiments. Cells incubated in medium, pH 7·4, containing 1μCi/ml.[$^3$H]uridine, 1g./l. glucose. ●——● anaerobic, 2 × $10^{-4}$ M Synkavit; ○——○ aerobic, 2 × $10^{-4}$ M Synkavit; ▲——▲ anaerobic, 2 × $10^{-5}$ M Synkavit; △——△ aerobic, 2 × $10^{-5}$ M Synkavit.

ably by treatment with Synkavit. Thus in both these cases, both at physiological and higher pH, treatment of the cells with Synkavit caused a specific release of nucleoside or base, and not a general leakage of all cellular components.

*Analysis of the ASF*

(a) *Labelled adenosine as precursor.*—Analysis of the ASF directly on DEAE-cellulose showed the presence of eight radioactive peaks, many of which correlated with the normal components (Fig. 8(b)). These were identified by chromatography with known compounds, except for the first peak of activity to be eluted from the column (peak 1) which may correlate with the small peak in the absorbance profile in that region, or may represent the active ribose remaining after cleavage of inosine to give the hypoxanthine which was then released into the medium. The results of the analyses of various ASF's isolated after increasing incubation times are given in Fig. 9(a). It is clear that the precursor adenosine was very rapidly metabolised, and therefore conclusions about the transport of adenosine cannot be drawn. However, the ADP activity quickly became stable, whilst the ratio of the activities of ATP and AMP fluctuated markedly, yet in a manner which maintained their sum approx. constant.

The effect of Synkavit on these processes is shown in Fig. 8(a) and 9(b). Two types of effect were consistently observed: first, a considerable decrease in the activity of ATP and, to a lesser extent, ADP and also of NAD+ (this result is not included in Fig. 9 for the sake of clarity). Secondly, a five-fold increase in the

FIG. 8.—Effect of Synkavit on nucleotide distribution in ASF. The cells were incubated for 90 minutes, aerobically at pH 7·4, the medium containing 1g./l. glucose, 0·5 μCi/ml.[$^3$H] adenosine. The ASF's were isolated and analysed on DEAE-cellulose. (a) treated $1 \cdot 5 \times 10^{-4}$ M Synkavit, (b) control. ---- activity, ——— absorbance at 254 mμ. Abscissa: tube number (3·7 ml./tube). Key: I, inosine; Ad, adenine; IMP, inosine-5′-monophosphate; A, adenosine.

activity of inosine occurred, together with a very large increase in the activity of IMP. Thus the control cells showed a barely detectable level of activity in the form of IMP, whereas after 75 minutes in the case of the treated cells this level rose to 40% of the total (cf. Fig. 8(a) and (b)). The activities associated with adenine, AMP and peak 1, on the other hand, were affected only slightly.

Essentially similar conclusions may be drawn from parallel measurements of the u.v. absorbance of these various components (Fig. 8). The amount of ATP was reduced very considerably after treatment of the cells with Synkavit, whereas IMP accumulated which was particularly noticeable by the change in absorbance maximum in that region from 260 mμ to 250 mμ.

FIG. 9.—(a) Variation with incubation time of the distribution of activity within the ASF components; (b) the effect of Synkavit on this distribution, expressed as ratio of activity associated with a given fraction after treatment to that of control. Incubation conditions as Fig. 8. ▲——▲ ATP, △——△ ADP, ●——● AMP, ×——× AMP + ATP, ○——○ inosine, ■——■ adenine, □——□ peak 1.

FIG. 10.—Incubation of cells with [$^3$H]uridine; distribution of activity in the ASF. Cells incubated in medium pH 7·4, containing about 1$\mu$Ci/ml.[$^3$H]uridine, and 1g./l. glucose, anerobically. ———— control; – – – – treated 2 × $10^{-4}$ M Synkavit. Key: U, uridine. 3·7 ml. fractions collected.

(b) *Labelled uridine as precursor.*—Fig. 10 shows the active components of the ASF resolved by chromatography on DEAE-cellulose when labelled uridine was used as precursor. The various peaks were characterised as described previously. It is not known with certainty whether the peak of activity at tube 300 is significantly shifted from the position of UTP. However, it was interesting to find that the labelling scheme favoured UDPG in preference to UDP. Again after treatment with Synkavit, two main effects were observed: a decrease in the activity of the nucleotides and an accumulation of nucleoside. For example, in one *anaerobic* experiment treatment of the cells with $10^{-4}$ M Synkavit altered the activities of

FIG. 11.—Analysis of nucleic acids on MAK column. ——— absorbance at 260 mμ; – – – – activity 0·8 ml. eluent. ↑ : commencement of 1% ammonia gradient. The peak of activity, $q_2$, is best resolved as a distinct peak at 30–45 minutes after a pulse labelling, but otherwise forms a shoulder to $q_1$, disappearing after about 1 hour. Cells were incubated for 45 minutes with [$^3$H]uridine, and nucleic acids extracted, added to column and eluted with a salt gradient as described in Methods. 3·2 ml. fractions collected.

uridine, UMP, UDPG and "UTP" in the ratios: 1·6, 0·25, 0·65 and 0·43 respectively. Thus, even under anaerobic conditions, inhibition of synthetic phosphorylation reactions occurred, with accumulation at the nucleoside level.

(c) *Labelled thymidine as precursor.*—Similar analyses of the ASF in the case when labelled thymidine was used as precursor showed that the activity in the ASF was associated entirely with thymidine, TMP, TDP and TTP. After a 90 minute incubation with $10^{-4}$ M Synkavit, the cellular activities of these compounds were altered in the ratios 1·1, 0·5, 0·2 and 0·15 respectively. Thus the reduction in nucleoside triphosphate activity was also observed with a specific precursor for DNA.

### *Analysis of the nucleic acids*

Preliminary analysis of the nucleic acids on columns of MAK showed that they could be resolved into various components (Fig. 11), as described by other workers (e.g. Ellem and Sheridan, 1963). Further, treatment of the cells with $10^{-4}$M Synkavit did not inhibit the synthesis of any component of the nucleic acids specifically. Thus after 45 minutes incubation with labelled uridine, the activities of various nucleic acid components of the treated cells were reduced in the ratios: sRNA, 0·4; "DNA", 0·6; rl RNA, 0·45; and a RNA, 0·3, relative to the controls. However, the result regarding DNA is subject to greater possible error since the specific activity was very low, and furthermore may not represent true DNA synthesis since the precursor uridine was specifically labelled in the 5 position of the pyrimidine nucleus.

Nevertheless, these results do suggest that the effects described previously on the cellular nucleoside triphosphate levels are the main cause of the observed reduction in synthesis of nucleic acids. In any case, the results described in this section must be interpreted in terms of the dependence of the synthetic routes of the various nucleic acid components on ATP and related triphosphates.

*Transplantation experiments*

The possibility was considered that the effects described above may have been a consequence of cell death caused by Synkavit. To check this point, control cells which were incubated at 37° C. for 30 minutes at pH 7·3 and treated cells incubated similarly with $1{\cdot}5 \times 10^{-4}$M Synkavit were separately injected into groups of mice ($10^7$ cells/mouse in 0·5 ml.). Each mouse was weighed daily until a 5 g. increase in weight occurred within a space of 3 days (work in this laboratory has shown that this statistic is the most reliable criterion of ascitic tumour growth (G. DiVita, unpublished work)). No significant differences were found between the control and treated groups in growth of the tumour. Furthermore, cells treated with Synkavit in the range of concentrations used in these experiments did not stain with eosin, except at extreme pH. It may be concluded, therefore, that the observed biochemical changes caused by Synkavit are reversible, and that cell death does not occur under these conditions.

### DISCUSSION

It is evident from the results presented in this paper that treatment of Ehrlich ascites cells *in vitro* with $10^{-5}$ M Synkavit reduces the rate of synthesis of nucleic acids from exogenous nucleosides by reducing the rate of their incorporation into the intracellular nucleoside triphosphates. This confirms earlier work using an enzymic method of estimation of cellular ATP, which was possibly subject to some interference by Synkavit (Chipperfield and Marrian, 1962). However, the present work shows that the effect is observed with three ribo-nucleosides and also with thymidine. Furthermore, the reduction of incorporation into nucleoside triphosphates is paralleled by a reduction in their total amounts, and leads to an accumulation of nucleoside within the cell which is subsequently released into the medium or degraded. This loss of material from the cell may be related to the state of oedema which develops after treatment with Synkavit (Hughes and Simon-Reuss, 1953). Moreover, treatment with Synkavit has been shown to have no effect on the subsequent growth characteristics of the cells after innoculation, and thus the biochemical effects described are essentially reversible and are not due to cell death.

The present work provides information as to the conditions under which these effects occur. Most critical is the pH dependence which has been discussed earlier. Secondly, the effect depends on the presence of glucose in the medium, which may be expected in view of the effect on the nucleoside triphosphate levels, since their synthesis is linked to glucose metabolism. The slight effect observed in the absence of glucose from the medium is probably explained by the small glucose pool remaining in the cell.

It will be noted that these results differ in some respects from those obtained by Marrian (1959). He found that the incorporation of labelled formate and glycine into DNA was not reduced by treatment with Synkavit, unlike the incorporation into RNA, and that the specific activity of the acid-soluble fraction was not affected, although its u.v. absorbance increased significantly. However, in his method of fractionating the cells, the ascitic fluid and cells *together* were treated with acid to give an acid-soluble fraction, which, in the experiments reported in this paper, is equivalent to the medium and ASF combined. Thus the discrepancy between the results at this point is more apparent than real. The fact that Marrian did not detect any effect on incorporation into DNA remains at variance with the results described in this paper, although it must be noted that his effects generally were smaller and that his experiments were performed *in vivo*, studying a more complex biochemical pathway. Moreover, Gronow (1963) showed that the use of formate as precursor introduced complications: the increase in absorbance of the acid-soluble fraction (by Marrian's method of isolation) occurred after treatment with formate alone, or with large doses of inorganic phosphate, but was not observed after treatment with Synkavit unless formate was *also* given.

The mechanism whereby treatment with Synkavit causes reduction in the levels of the nucleoside triphosphates is not known with certainty. Studies of the enzymic dephosphorylation of Synkavit implicate menadiol as the initial product which is quickly oxidised to menadione (Ramasarma, *et al.*, 1959, although under extreme alkaline conditions this may be converted to the deep orange-coloured 2-methyl-3-hydroxy-1,4-napthaquinol (Hollocher and Weber, 1962). Many workers have reported that menadione uncouples oxidative phosphorylation (e.g. Chem and Dallam, 1963), and the mechanism of this effect has been elucidated. Conover and Ernster (1962) have shown that menadione interacts with the cytochrome chain at the point of ubiquinone and thereby by-passes one phosphorylation site (see also Slater *et al.*, 1961). This alternative route is meditated by an enzyme, DT diaphorase (E.C. 1.6.5.2), which catalyses oxidation of both NADH and NADPH (Ernster, Danielson and Ljungeren, 1962). Thus the pentose phosphate shunt pathway may be linked at this point *via* NADPH. In fact, Wenner, Hackney and Moliterno (1958) showed that in mouse ascites cells (in contrast to mouse liver cells), metabolism of glucose by the shunt route occurred anaerobically as well as aerobically. Moreover, menadione stimulated glucose oxidation *via* this pathway. However, in brain tissue Hoskin (1960) obtained evidence that diversion of glucose metabolism into the shunt by menadione was not related to its uncoupling action.

Further work (Tiedemann *et al.*, 1958) investigating glycolysis in mouse ascites cells showed that menadione inhibited aerobic but not anaerobic glycolysis at molarities less than $10^{-4}$. The latter effect was dependent on flushing the apparatus *before* the menadione was added with nitrogen, and was not observed if a 5% $CO_2$ in $N_2$ mixture was used. Since the buffer used was a bicarbonate type, some pH change may have been involved. The result may explain the reduction by Synkavit in the rate of acid production when the cells were incubated with glucose, which was observed in the experiments reported in this paper. Further, the inhibition of glycolysis may involve the markedly SH-dependent enzyme, glyceraldehyde-3-phosphate dehydrogenase (E.C. 1.2.1.12), since menadione is known to react with –SH groups (Friedmann, Marrian and Simon-Reuss, 1948).

Thus all the available data indicate that the situation is complex. Furthermore, it is difficult to establish conclusively whether some metabolite of Synkavit inhibits the synthesis of ATP directly or whether it stimulates its breakdown. Indeed, the stimulation of mitochrondrial ATPase activity is a consequence of the uncoupling action of menadione (Schulz and Goss, 1956). Experiments have been designed to try to elucidate this point, and are in progress at the time of writing.

Finally, the significance of these findings for radiotherapy may be briefly discussed. Synkavit has been used both as a radiosensitising agent and, in tritiated form, as a radioactive drug (Mitchell and Marrian, 1965), since it is selectively incorporated into many tumour cells (Mitchell *et al.*, 1963). It has now been established that this incorporation is pH dependent, presumably involving an alkaline phosphatase in or near the cell membrane. However, especially in tumours growing under anoxic conditions, the pH may be less than 7·0 (Ashby, 1966) which would prevent extensive uptake by this enzyme. Nevertheless, Synkavit, once incorporated, would be effective since it has been shown to act anaerobically. This is a particularly useful finding since lethal radiation damage is known to be less severe under anoxic conditions. Since ATP constitutes the major intermediate energy source in the cell, and since the ATP/ADP ratio plays an important role in the regulation of cell metabolism, the effects of Synkavit may be expected to make the cell more sensitive to damage, and in particular, to radiation damage. That the radiosensitising properties of Synkavit and the effects described in this paper are related may be suggested by the finding in this laboratory that menadione acts as a radiosensitiser of ascites cells anaerobically, as determined by the effects of radiation and Synkavit on the growth characteristics of an inoculum of ascites cells.

### SUMMARY

The effect of the radiosensitising agent, Synkavit, (2-methyl-1,4-napthaquinol bis disodium phosphate) on the synthesis of nucleic acids in Ehrlich ascites cells *in vitro* has been investigated. It has been shown that $10^{-5}$ M Synkavit reduces the incorporation of labelled ribo-nucleosides and thymidine into RNA and DNA respectively, and into the acid-soluble nucleotide pool, with concomitant release of nucleoside (or, with adenosine as precursor, hypoxanthine) into the medium. These effects are only observed fully when the pH of the medium is greater than 7·0, and when it contains glucose, but they are not dependent on oxygen.

By chromatography of the acid-soluble nucleotide pool on DEAE-cellulose, it has been shown that both the incorporation of labelled nucleosides into their respective triphosphates and the total amounts of cellular nucleoside triphosphates are reduced by treatment of the cells with Synkavit, with a consequent accumulation at the nucleoside level. Evidence is presented to show that the synthetic processes are inhibited leading to stimulation of degradative pathways. However, pretreatment of the ascites cells *in vitro* with Synkavit does not alter their growth characteristics on subsequent innoculation.

The possible explanations and significance of these findings for radiotherapy are discussed.

I should like to thank Professor J. S. Mitchell, F.R.S., and Dr. D. H. Marrian for their constant interest and encouragement concerning the work reported in this paper, and to many members of the Department with whom I have had many useful discussions, particularly Dr. Valerie Fisher. I am also indebted to Mr. E. A. King who performed the transplantations necessary for the experiments, and to Dr. G. DiVita for the original idea behind the incubation apparatus. Finally, I am indebted to the Medical Research Council for the tenure of a Scholarship for Training in Research Methods.

### REFERENCES

ASHBY, B. S.—(1966) *Lancet*, ii, 312.

CARTER, C. E.—(1950) *J. Am. chem. Soc.*, **72**, 1466.

CHEM, L. H. AND DALLAM, R. D.—(1963) *Fedn Proc. Fedn Am. Socs exp. Biol.*, **22**, 405.

CHIPPERFIELD, BARBARA AND MARRIAN, D. H.—(1962) *Br. J. Cancer*, **16**, 460.

CONOVER, T. E. AND ERNSTER, L.—(1962) *Biochim. biophys. Acta.*, **58**, 189.

DAVEY, C. L.—(1962) *Biochim. biophys. Acta.*, **61**, 538.

ELLEM, K. A. D. AND SHERIDAN, J. W.—(1963) *Biochem. biophys. Res. Commun.* **13**, 61.

ERNSTER, L., DANIELSON, L. AND LJUNGEREN, M.—(1962) *Biochim. biophys. Acta.*, 58, 171.

FLECK, A. AND BEGS, D.—(1965) *Biochim. biophys. Acta.*, **108**, 333.

FRAENKEL-CONRAT, H., SINGER, B. AND TSUGITA, A.—(1961) *Virology*, **14**, 54.

FRIEDMANN, E., MARRIAN, D. H. AND SIMON-REUSS, IRMELIN—(1948) *Br. J. Pharmac. Chemother.*, **3**, 335.

GILL, D. M.—(1967) *Int. J. appl. Radiat. Isotopes*, **18**, 393.

GRONOW, M.—(1963) Ph.D. Thesis, Cambridge University.

HOLLOCHER, T. C. AND WEBER, M.M.—(1962) *Nature, Lond.*, **195**, 247.

HOSKIN, F. C. G.—(1960) *Archs Biochem. Biophys.*, **91**, 43.

HUBINSKI, H., KOCH, G. AND DREES, O.—(1962) *Biochim. biophys. Acta.* **61**, 332.

HUGHES, A. AND SIMON-REUSS, IRMELIN—(1953) *Br. J. Cancer*, **7**, 142.

MANDELL, J. D. AND HERSHLEY, A. S.—(1960) *Analyt. Biochem.*, **1**, 66.

MARRIAN, D. H.—(1959) *Br. J. Cancer*, **13**, 461.

MITCHELL, J. S., KING, E. A., MARRIAN, D. H. AND CHIPPERFIELD, BARBARA—(1963) *Acta radiol.*, **1**, 321.

MITCHELL, J. S. AND MARRIAN, D. H.—(1965) 'Biochemistry of Quinones', London (Academic Press Inc.) p. 503.

NICOLAU, C., KORNER, O. AND CRISTEA, A.—(1966) *Studia biophysica*, **1**, 59.

RAMASARMA, T., SCRINAVASAN, N. G., SRIPATHI, C. E. AND SIVARAMAKRISHNAN, V. B.—(1959) *Enzymologia*, **21**, 133.

SCHULZ, A. R. AND GOSS, H.—(1956) *Biochim. biophys. Acta.*, **21**, 578.

SLATER, E. C., COLPA-BOONSTRA, J. P. AND LINKS, J.—(1961) 'Quinones in Electron Transport', London (Churchill) p. 161.

TIEDEMANN, V. H., RISSE, H. J. AND BORN, J.—(1958) *Z. Naturf.*, **13**, 657.

WENNER, C. E., HACKNEY, J. H. AND MOLITERNO, F.—(1958) *Cancer Res.*, **18**, 1105.